# Supplementary material for: Effects of nanoscale zinc oxide treatment on growth, rhizosphere microbiota, and metabolism of Aconitum carmichaelii
Source: PeerJ. 2023 Oct 18;11:e16177. doi: 10.7717/peerj.16177 (PMC10590109; doi:10.7717/peerj.16177)
Supplement: Table S1 [file peerj-11-16177-s009.docx]

**Table S1 Up- and down-regulated metabolites in lateral roots.**

| **NO.** | **Compounds** | **HMDB.ID** | **VIP** | **FC** | **log2(FC)** | **Trend** |
| --- | --- | --- | --- | --- | --- | --- |
| BGI1271 | D-Mannosamine | #N/A | 1.633729 | 10.583 | 3.4037 | up |
| BGI0204 | Epigoitrin | #N/A | 1.867012 | 10.256 | 3.3585 | up |
| BGI1295 | Maleamic Acid | #N/A | 1.932121 | 9.6057 | 3.2639 | up |
| BGI0831 | D-(+)-Glucosamine | HMDB0001514 | 1.623788 | 8.4158 | 3.0731 | up |
| BGI1389 | Creatinine | HMDB0000562 | 1.974651 | 7.7529 | 2.9547 | up |
| BGI0836 | D-Proline | HMDB0003411 | 1.914085 | 7.6341 | 2.9325 | up |
| BGI0426 | 2-Pyrrolidinecarboxylic acid | HMDB0000162 | 1.830881 | 7.1692 | 2.8418 | up |
| BGI1509 | DL-Mevalonolactone | #N/A | 1.762755 | 7.1605 | 2.8401 | up |
| BGI1485 | Metformin | #N/A | 1.926978 | 5.8714 | 2.5537 | up |
| BGI0786 | Agmatine | HMDB0001432 | 1.955606 | 5.2616 | 2.3955 | up |
| BGI1119 | Serotonin | HMDB0000259 | 1.926793 | 4.7732 | 2.255 | up |
| BGI0865 | N-Methyltyramine | HMDB0003633 | 1.624173 | 4.4704 | 2.1604 | up |
| BGI1572 | Spinosin | #N/A | 1.851059 | 3.6263 | 1.8585 | up |
| BGI1530 | S-Methyl-L-cysteine sulfoxide | #N/A | 1.554506 | 3.3959 | 1.7638 | up |
| BGI1070 | Dehydroascorbic acid | HMDB0001264 | 1.996279 | 2.9427 | 1.5571 | up |
| BGI0960 | 14-Benzoylaconine | #N/A | 1.708522 | 2.8659 | 1.519 | up |
| BGI1235 | Trigonelline | HMDB0000875 | 1.559717 | 2.5744 | 1.3642 | up |
| BGI0994 | Bengenin | #N/A | 1.59517 | 2.3542 | 1.2353 | up |
| BGI1465 | Shanzhiside methyl ester | #N/A | 1.664437 | 2.3285 | 1.2194 | up |
| BGI0896 | L-Alanyl-L-glutamine | HMDB0028685 | 1.977197 | 2.2754 | 1.1861 | up |
| BGI0293 | Tranexamic Acid | HMDB0014447 | 1.900233 | 2.16 | 1.111 | up |
| BGI0733 | Orientin | HMDB0030614 | 1.996175 | 2.14 | 1.0976 | up |
| BGI1748 | Rosamultin | #N/A | 1.615759 | 2.0614 | 1.0436 | up |
| BGI1689 | Rehmannioside C | #N/A | 1.668368 | 0.45946 | -1.122 | down |
| BGI1063 | Ribitol | HMDB0000508 | 1.683512 | 0.44817 | -1.1579 | down |
| BGI1621 | L-Arabitol | HMDB0001851 | 1.683572 | 0.44534 | -1.167 | down |
| BGI1376 | Limonene | HMDB0004321 | 1.610267 | 0.4216 | -1.246 | down |
| BGI1375 | (R)-(+)-Limonene | HMDB0003375 | 1.526507 | 0.41846 | -1.2568 | down |
| BGI1560 | Cyanidin-3-O-glucoside chloride | #N/A | 1.576324 | 0.40528 | -1.303 | down |
| BGI1831 | gamma-Terpinene | HMDB0005806 | 1.688241 | 0.39151 | -1.3529 | down |
| BGI1242 | Hordenine | HMDB0004366 | 1.517187 | 0.28858 | -1.793 | down |
| BGI1004 | Astragalin | HMDB0037429 | 1.555047 | 0.20415 | -2.2923 | down |
| BGI1236 | Cynaroside | HMDB0035588 | 1.606359 | 0.1933 | -2.3711 | down |
| BGI0504 | Kaempferol-3-O-rutinoside | #N/A | 2.039387 | 0.18474 | -2.4364 | down |
| BGI0611 | Glucoraphanin | HMDB0038404 | 1.5576 | 0.15883 | -2.6544 | down |
| BGI0800 | L-(+)-Selenomethionine | HMDB0003966 | 1.692547 | 0.12455 | -3.0052 | down |
| BGI1224 | Scopolin | #N/A | 1.720721 | 0.10371 | -3.2694 | down |
| BGI0990 | Boldine | #N/A | 1.794432 | 0.015558 | -6.0062 | down |
