# Supplementary material for: Effects of nanoscale zinc oxide treatment on growth, rhizosphere microbiota, and metabolism of Aconitum carmichaelii
Source: PeerJ. 2023 Oct 18;11:e16177. doi: 10.7717/peerj.16177 (PMC10590109; doi:10.7717/peerj.16177)
Supplement: Table S2 [file peerj-11-16177-s010.docx]

**Table S2 Mean proportion of Hierarchy level 2 within bacteria communites under CK and ZnO NPs treatments. P<0.05.**

| Class2 | ZnONPs: mean rel.freq.(%) | CK: mean rel.freq.(%) | p-values | 95.0% lower CI | 95.0% upper CI |
| --- | --- | --- | --- | --- | --- |
| Drug resistance: Antimicrobial | 0.804100459 | 0.820366033 | 0.000369 | -0.017919919 | -0.01461123 |
| Immune diseases | 0.032450865 | 0.028119821 | 0.001956 | 0.003106039 | 0.005556049 |
| Endocrine and metabolic diseases | 0.221787429 | 0.227776109 | 0.002399 | -0.007433244 | -0.004544116 |
| Signaling molecules and interaction | 0.031155762 | 0.032863692 | 0.002767 | -0.002241349 | -0.001174511 |
| Lipid metabolism | 2.52944549 | 2.475335716 | 0.0028 | 0.036173692 | 0.072045857 |
| Transport and catabolism | 0.304378611 | 0.281426808 | 0.003573 | 0.016694892 | 0.029208713 |
| Carbohydrate metabolism | 8.917273273 | 8.74982034 | 0.005337 | 0.115878729 | 0.219027138 |
| Cell growth and death | 0.689293397 | 0.712397008 | 0.005345 | -0.031395145 | -0.014812077 |
| Translation | 2.739954526 | 2.867628322 | 0.006004 | -0.181413969 | -0.073933625 |
| Transcription | 0.120215445 | 0.124533045 | 0.006175 | -0.006210038 | -0.002425163 |
| Metabolism of cofactors and vitamins | 3.993655421 | 4.041155829 | 0.006266 | -0.068956505 | -0.026044312 |
| Energy metabolism | 4.369580402 | 4.423775956 | 0.006348 | -0.07551322 | -0.032877888 |
| Biosynthesis of other secondary metabolites | 0.971727884 | 0.938673635 | 0.00673 | 0.020716447 | 0.045392051 |
| Folding, sorting and degradation | 1.369871479 | 1.411077134 | 0.008339 | -0.061427863 | -0.020983447 |
| Digestive system | 0.030510783 | 0.027445399 | 0.015718 | 0.001519101 | 0.004611667 |
| Environmental adaptation | 0.137230573 | 0.139749708 | 0.016209 | -0.003972605 | -0.001065664 |
| Membrane transport | 3.354061305 | 3.258319342 | 0.017732 | 0.036237904 | 0.155246022 |
| Nervous system | 0.183715101 | 0.178509795 | 0.018445 | 0.002187418 | 0.008223194 |
| Nucleotide metabolism | 2.903861262 | 2.946244441 | 0.01859 | -0.067399282 | -0.017367076 |
| Replication and repair | 2.314978834 | 2.363016828 | 0.019368 | -0.074796826 | -0.021279163 |
| Aging | 0.438946976 | 0.448118723 | 0.022442 | -0.015175353 | -0.003168141 |
| Metabolism of terpenoids and polyketides | 1.265445144 | 1.253564933 | 0.023283 | 0.003973642 | 0.019786779 |
| Endocrine system | 0.603491224 | 0.591855832 | 0.025758 | 0.003521849 | 0.019748935 |
| Circulatory system | 0.031771474 | 0.036312644 | 0.028298 | -0.007560478 | -0.001521862 |
