# Supplementary figures and images for: Effects of nanoscale zinc oxide treatment on growth, rhizosphere microbiota, and metabolism of Aconitum carmichaelii
Source: PeerJ. 2023 Oct 18;11:e16177. doi: 10.7717/peerj.16177 (PMC10590109; doi:10.7717/peerj.16177)

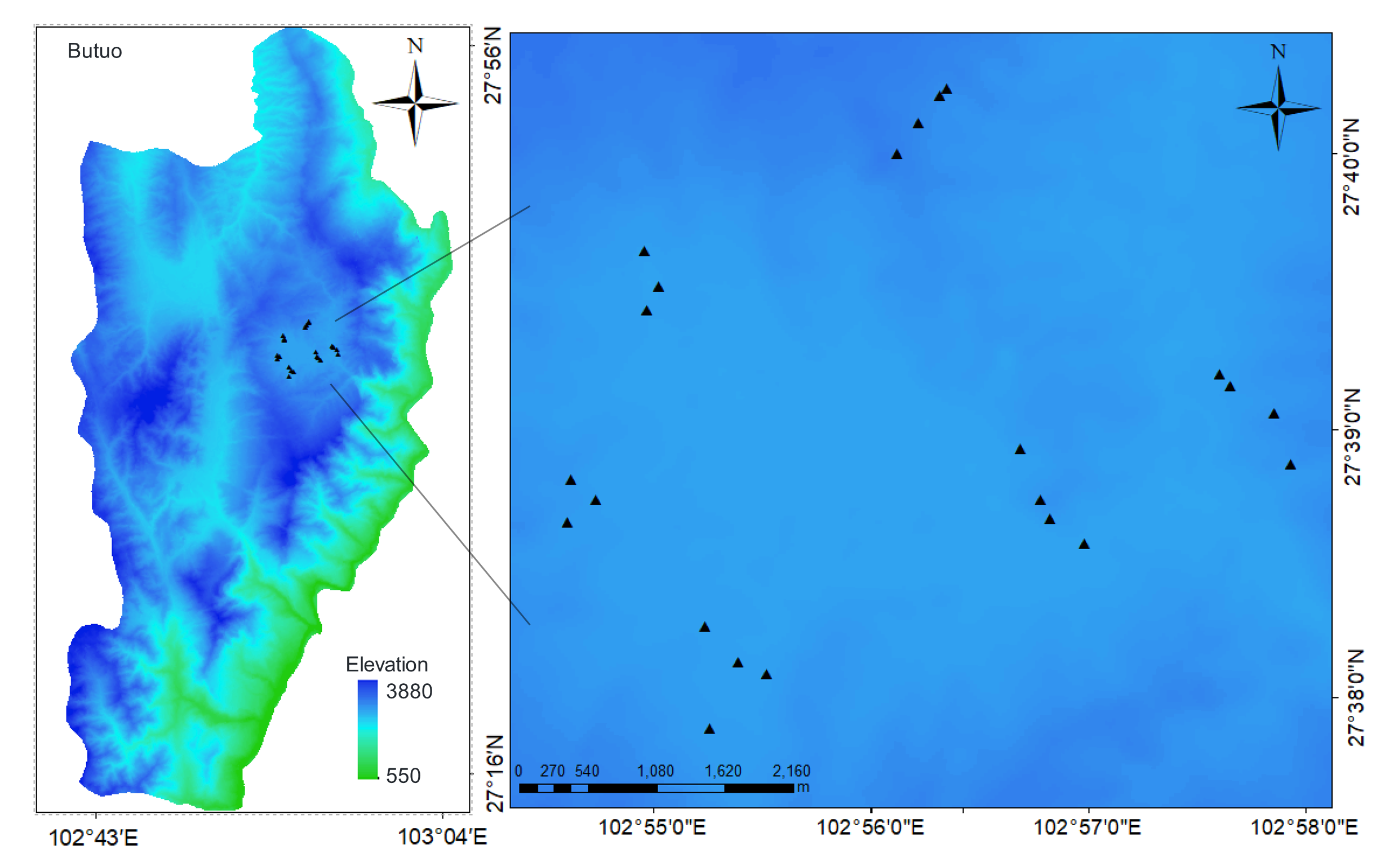

Supplement: Figure S1 [file peerj-11-16177-s011.png]

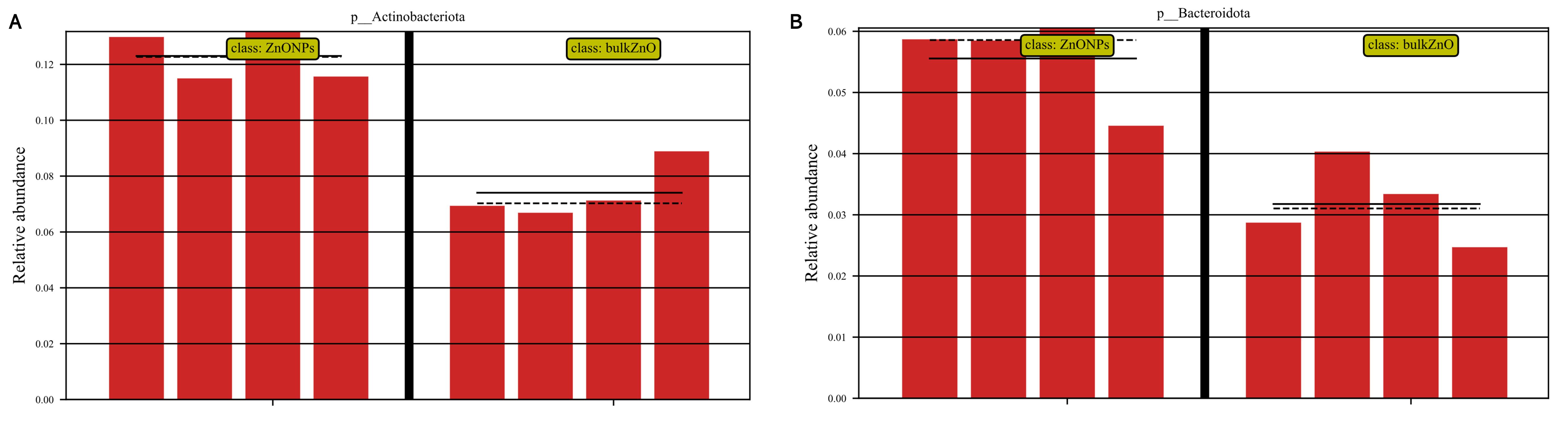

Supplement: Figure S2 [file peerj-11-16177-s012.png]
